# The swelling performance of raw and modified bentonite of geosynthetic clay liner as the leachate barrier exposed to the synthetic E-waste leachate

**DOI:** 10.1016/j.heliyon.2022.e11937

**Published:** 2022-12-02

**Authors:** Maryam Roshan Mooshaee, MohammadReza Sabour, Ebad Kamza

**Affiliations:** a Civil Engineering Faculty, K. N. Toosi University of Technology, Tehran, Iran; b Geotechnical Engineer, Tehran, Iran

**Keywords:** Geosynthetic Clay Liners, CarboxyMethyl Cellulose, Electrical and electronic waste, Free swell index, Response Surface Method (RSM)

## Abstract

Heavy metals are prevalent in electrical and electronic waste. The sealing of this type of waste disposal site is critical due to the existence of toxic materials. In this regard, Geosynthetic Clay Liners (GCLs) are widely used as one of the most common engineered barriers at disposal sites. Recently, attention has been drawn to modifying the bentonite of GCL with polymers to improve barrier performance against leachate. The aim of this study was to evaluate the swelling performance of the raw and modified GCL with a hydrophilic anionic polymer called CarboxyMethyl Cellulose (CMC) with weight percentages of 8, 10, and 12 of dried bentonite against synthetic heavy metals’ leachate, containing copper and zinc, simultaneously and separately, based on ASTM D5890. It was found that adding this polymer could improve the swelling rate of the GCLs. The optimum CMC rate for modified GCLs exposed to the cationic solutions, including copper and zinc, was estimated at 10%. It could also be noted that the swell index of both natural and modified bentonites against solutions, including two cations of copper and zinc, simultaneously, was more sensitive to the changes in zinc ion concentration versus copper metal concentration.

## Graphical abstract

**Figure:**
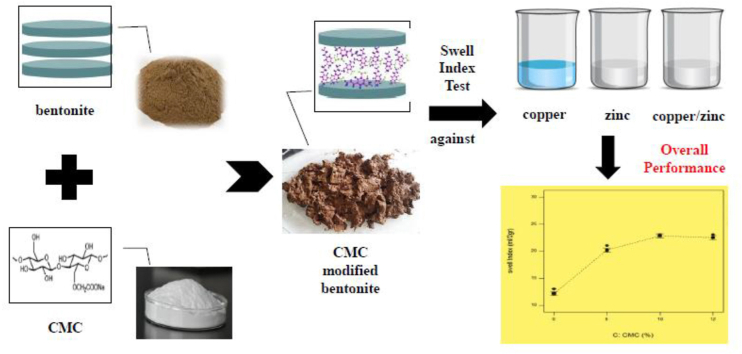

## 1 Introduction

Electrical and electronic waste (E-waste) has recently become a global concern due to its high volume of toxic heavy metals, such as copper, zinc, cadmium, mercury, magnesium, nickel, arsenic, chromium, and iron (Adaramodu et al., 2012; Robinson, 2009). This type of waste can trigger numerous serious diseases in humans, such as DNA damage, cancer, and some allergies, especially who are living in areas near landfills and recycling places (Dutta and Mishra, 2016; Grant et al., 2013; Tabelin et al., 2018). Additionally, compared to earlier findings, the quantities of heavy metals originating from E-waste have grown, particularly in incineration facilities (Ohajinwa et al., 2018). Groundwater quality and the environment are both threatened by the migration of heavy metals into the soil (Kaoser, Barrington, Elektorowicz and Wang, 2004b). Thus, additional research is needed to prevent heavy metals from entering into the environment and minimize their irreversible effects.

Geosynthetic Clay liners (GCLs) have been employed as leachate barriers in a variety of geotechnical and environmental applications such as landfills, water and sewage pools, and so forth due to their low hydraulic conductivity, high swelling potential, and ability to self-healing (Kaoser et al., 2004b; Sato et al., 2017). GCLs consist of two geotextile layers and a thin layer of bentonite, which determines barrier performance against leachate (Rowe, 2014). Sodium cations in bentonite can interact with water. The volume of water molecules attached to bentonite surfaces, denoted by diffuse double layer (DDL), forms tortuous flow paths and acts as an impediment to permeant solutions. If this volume increases, not only will the empty spaces decrease in the bentonites to the permeant solutions but also the distance between bentonite layers and its swelling potential will increase (De Camillis, Di Emidio, Bezuijen and Flores, 2016; Katsumi et al., 2007; Salemi et al., 2018). The high swelling rate of bentonite can lead to a decrease in bentonite permeability (Chen et al., 2018). Various factors influence the bentonite performance, such as the bentonite structure, permeating solutions properties, the cation exchange capacity, temperature, the specific surface area of clay, and so on (Kong et al., 2017). For example, leachate with high ionic concentrations can negatively impact bentonite performance. In other words, the swelling rate of bentonite decreases with an increase in the ionic concentrations of leachate. The reason behind this matter is to reduce the distance between the bentonite layers due to pollutants' adsorption and cation exchange by the bentonite negative surfaces (De Camillis, Di Emidio, Bezuijen and Verastegui-Flores, 2018; Dutta and Mishra, 2016; Xue et al., 2012). Also, the results of chemical analyses indicate that with an increase in the ion concentration of leachate, the cation exchange in the bentonite ascends (Rosin-Paumier et al., 2011). It follows that more of a decrease in the layer spacing of the bentonite can be seen. Moreover, competition is created between the cations to participate in the cation exchange with the bentonite while exposed to positive valence pollutants, such as heavy metals. Cations properties have governed this competition, such as valences, ion hydration radius, and the presence of other ions in solutions (Kaoser et al., 2004b; Scalia IV and Benson, 2011). In terms of ion charge capacity, GCLs exposed to solutions, containing divalent or trivalent cations have higher hydraulic conductivity and lower swell index than GCLs against solutions, including monovalent cations of the same concentration or deionized water (Jo et al., 2001; Xue et al., 2012). If solutions have pollutants with equal valence conditions, the amount of adsorption is inversely related to the hydrated radius of the ions (Jo et al., 2001; Kaoser et al., 2004b). It means that the smaller the hydrated radius, the higher the adsorption rate. In addition, the rate of swelling of bentonite in the presence of solutions containing divalent or polyvalent cations is less than that of monovalent solutions with the same concentration (Dutta and Mishra, 2016). In terms of the presence of other ions in solutions, the adsorption rate of a specific ion can be descended by the existence of a stronger one in that solution. For instance, in liner exposed to the solutions, including Cu^2+^/Cd^2+^, and Cu^2+^/Pb^2+^, the presence of Cd^2+^ and Pb^2+^ ions strongly had a positive effect on Cu^2+^ adsorption by bentonite (Kaoser, Barrington, Elektorowicz and Wang, 2004a). Finally, the presence of different toxic substances in landfill leachate deteriorates the GCLs’ performance by affecting their adsorption capacity and hydraulic properties (Ray et al., 2021). This matter leads to endangering the environment and the groundwater. Hence, an understanding of pollutants behaviour in bentonite can play a vital role in barrier improvement and should be considered before preliminary implementation (Kaoser et al., 2004b).

**Figure 1:**
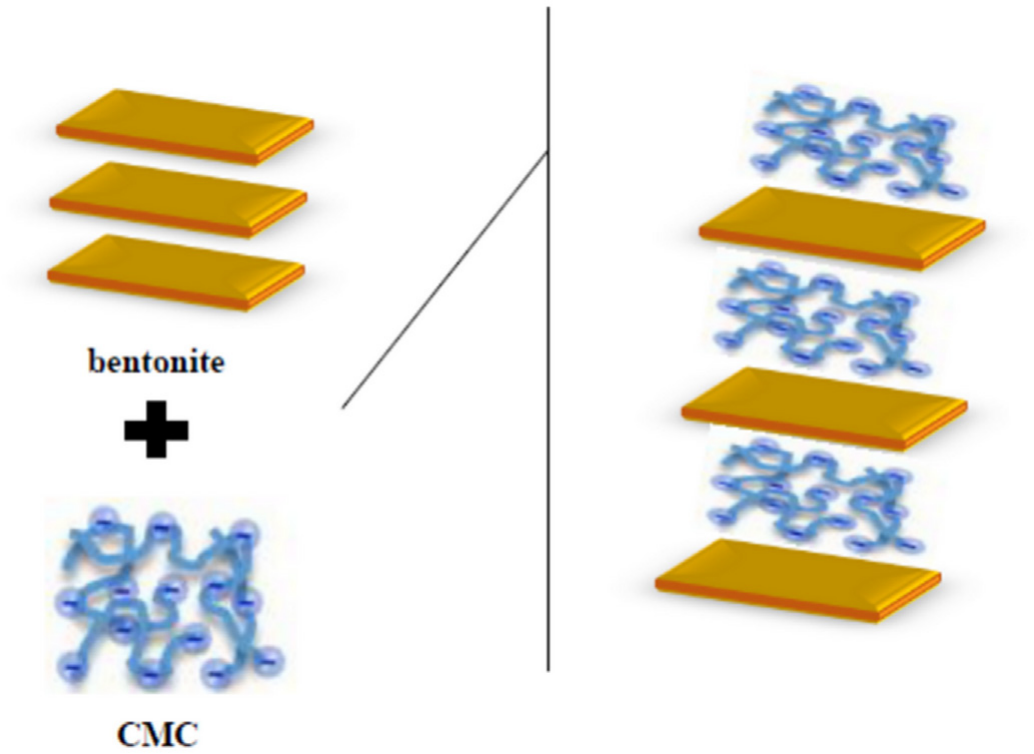
Schematic showing the polymer modified bentonite.

A modification method with additives, such as polymers, has been recently paid attention to in order to improve the GCLs’ performance and the bentonite properties against leachate (Faghihian and Nejati-Yazdinejad, 2009; Joshi and Srivastava, 2016; Kong et al., 2017; Kumararaja et al., 2018; Lo et al., 1997; Saeedi et al., 2018; Sato et al., 2017; Scalia et al., 2011; Xu et al., 2018). Figure 1 shows the schematic effects of the polymer content on the structure of the bentonite (del Mar Orta et al., 2020). In total, the polymer can enter the layer structure of the clay and this matter can increase the distance between bentonite layers and their specific surface area to ascend the adsorption rate of pollutants.

With the technological improvement, new adsorbents have entered the market in order to remove contaminants, such as anionic polymers, which have significant advantages, such as being non-toxic, stable, and having significant effects on the distance between the bentonite layers and then the bentonite permeability (Kong et al., 2017; Naderi et al., 2018). In fact, their negative charge can promote the barriers' performance, the number of trapped contaminants between the bentonite layers, and the cation exchange capacity because of an increase in the distance between the negative layers of the bentonite (Razakamanantsoa and Djeran-Maigre, 2016). Yet, a major concern is indicated that the bonds between polymers and the bentonites will be weaker and probably not have the same good function as the initial stage with the passing of time. Hence, further research is necessary to evaluate the modified GCLs’ performance with different polymers against leachates (Prabhu and Prabhu, 2018).

Cellulose, which is one of the anionic polymers, has been widely focused on due to its availability in nature, degradability, non-toxic, and less expensive. Among compounds containing cellulose, CarboxyMethyl Cellulose (CMC), which has hydrophilic functional groups such as hydroxyl (OH) and carbonyl (COOH), can provide maximum adsorption of water on clay particle surfaces, accumulating at an almost double swelling rate (Askari et al., 2016) due to its short chains (Amorim et al., 2007). As a result, CMC can increase the swell index of bentonite and reduce the barrier's permeability against water and pollutants (Amorim et al., 2007; Bohnhoff and Shackelford, 2015; Di Emidio, Mazzieri, Verastegui-Flores, Van Impe and Bezuijen, 2015; Di Emidio, Van Impe and Flores, 2011; Fan et al., 2018; Qiu and Yu, 2008; Tang et al., 2014).

Although E-waste is indicated as one of the main concerns throughout the world, to the writers' knowledge, no study has reported on the GCLs' performance exposed to E-waste leachates, including two heavy metals, such as copper and zinc, simultaneously. In addition, the bentonite modification method with polymers has been used to improve the barrier's performance against leachates because pollutants can deteriorate the GCLs' performance with the passing of time. While, this issue is still in the research stage. One of the main aims of this study was to investigate both natural and modified GCL's performance against binary solutions, including heavy metals such as copper (Cu^2+^) and zinc (Zn^2+^). For this matter, the swell index test, which is considered one of the most important parameters to evaluate the GCL's performance as a barrier, was conducted in this research. The results of this research can be a substantial help for engineers and designers to gain more knowledge about the effects of two dominant heavy metals in E-waste leachate, copper and zinc, on each other and on the GCL performance. The barrier performance improvement against toxic materials, the prevention of entering pollutants into the soil and groundwater, and the limitation of their dangerous effects while permeating were other important objectives behind this research. Yet, in order to be assured of using the safer barrier, other GCL test such as hydraulic conductivity in the short and long-term exposure to the considered synthetic and real leachate must be evaluated in the future. The real leachate can differ in results due to the existence of various ions in it. In addition, the chemical analysis of the passed leachate of the GCL helps to understand the rate of trapped pollutants in the bentonite of the barrier.

## 2 Materials

In this framework, the GCL performance with natural bentonite and CMC-modified bentonite exposed to the synthetic heavy metals leachates, including copper and zinc, was evaluated by nearly 100 swell index tests on the basis of ASTM D5890. The bentonite obtained from the GCL was modified by CMC with 8, 10, and 12% (dry mass of bentonite). In order to modify the bentonite, initially, CMC, which was dissolved in deionized water at 50 °C, was mixed in a mixer for 30–45 min to obtain a homogeneous mixture of the bentonite. Then, the resulting mixture was placed in an oven at 105 °C for 16 h. At the last step, the obtained CMC-bentonite was passed through sieve #200 according to the standard to prepare for the free swell index test (Alaskari and Teymoori, 2017; Benchabane and Bekkour, 2008; Di Emidio et al., 2011; Janssen et al., 2015; Menezes et al., 2010; Vryzas et al., 2019).

Deionized water (EC = 3 μs/cm) and synthetic heavy metals leachate, including copper, zinc, and copper/zinc, were considered the passing leachate. To prepare the passing solutions, 0.5, 1.5, 2.5, 3.5, and 4.5 g CuSO^4^.5H^2^O salt and 2.5, 3.5, 4.5, 5.5, and 6.5 g Zn(NO^3^)^2^ salt were dissolved in 1 L of Deionized water in order to yield the target amount of the proposed heavy metals in the solutions (Mazzieri et al., 2013). Moreover, the response surface method (RSM) at the Design-Expert software was used to determine specific concentrations of the expected pollutants to form mono- and bi-cationic solutions.

Table 1 lists the concentrations of used salts for the synthetic proposed solutions and their chemical properties, such as electrical conductivity (EC) and total dissolved solids (TDS). The aim of some repetitive concentrations was to ensure the accuracy of the results.

**Table 1:** Chemical properties and concentrations used for the permeating solution.

Solution	EC (μs/cm)	TDS (ppm)	Solution	EC (μs/cm)	TDS (ppm)
0.5 g Cu	357	181	Deionized Water	3	2
1.5 g Cu	820	406	2.5 g Cu + 2.5 g Zn	2570	1290
2.5 g Cu	1145	568	3.9 Cu + 5.9 g Zn	4720	2360
3.5 g Cu	1352	664	2.5 g Cu + 6.5 g Zn	4540	2270
4.5 g Cu	1578	789	0.5 g Cu + 4.5 g Zn	2990	1500
2.5 g Zn	1374	688	2.5 g Cu + 4.5 g Zn	3470	1750
3.5 g Zn	1878	945	1.1 g Cu + 3.1 g Zn	1945	972
4.5 g Zn	2830	1420	3.9 g Cu +3.1 g Zn	3310	1660
5.5 g Zn	3240	1710	1.1 g Cu + 5.9 g Zn	3870	1940
6.5 g Zn	3950	1980	4.5 g Cu + 4.5 g Zn	4180	2100

## 3 Test methods

### Free swell index

**Figure 2:**
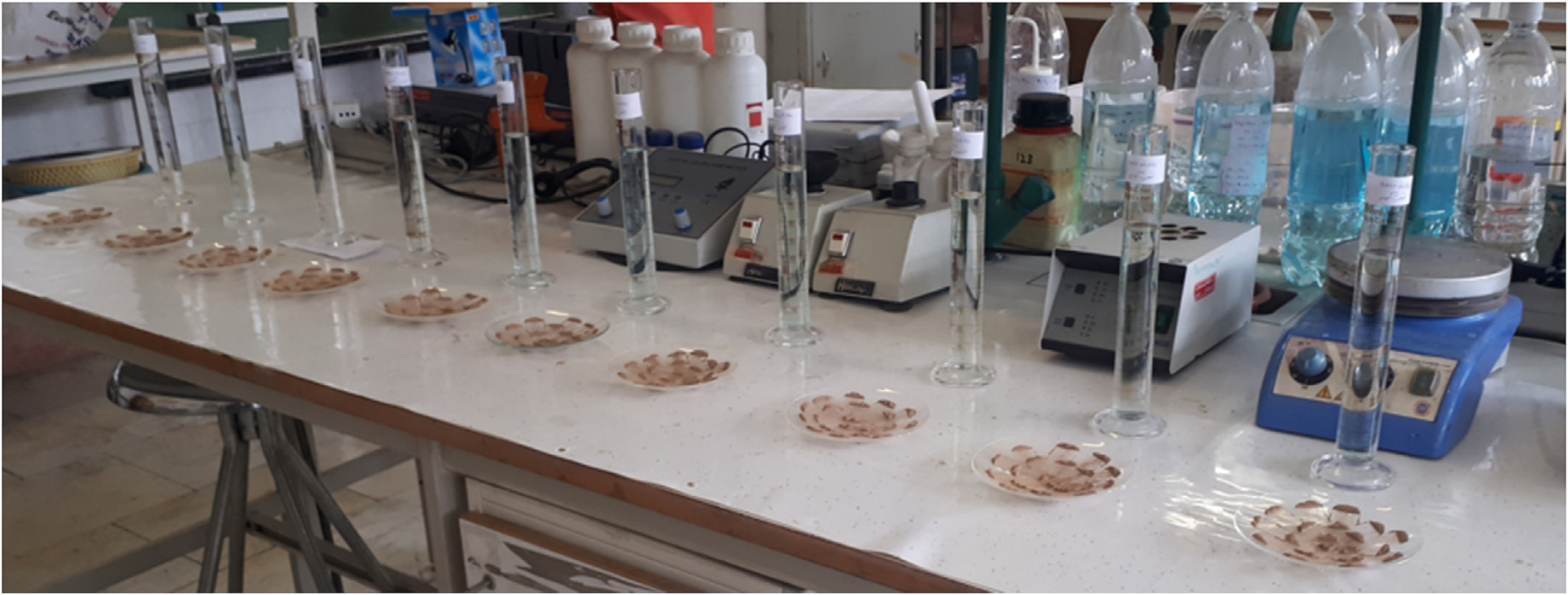
Sample preparation to conduct free swell index test.

Some specific tests can evaluate the GCLs' performance as the leachate barriers, such as hydraulic conductivity and swell index. The hydraulic conductivity test is a major measurement to investigate the barriers’ behaviour. Yet, due to being time-consuming the permeability test, the free swell index was conducted (Sato et al., 2017) on the basis of the ASTM D5890 standard (ASTM, 2003) in order to evaluate the swelling potential of bentonite and modified bentonite obtained from GCL. This experiment can investigate the effect of the proposed polymer on the swelling potential of the bentonite of the GCL in the presence of E-waste leachate, including heavy metals such as copper and zinc. According to this standard, in the first step, 2 g of the dry powdered raw and modified bentonite, which was removed from the oven, was gradually added to 90 ml of the solution poured into a 100 ml graduated cylinder. In the last step, the swelling height of the bentonite was determined after 16 h Figure 2 shows sample prepared to conduct the swell index test.

### Scanning Electron Microscopy

Scanning Electron Microscopy (SEM) was used to confirm the effect of the polymer content on the structure and the distance between the modified bentonite layers in terms of microscopic picture (Atigh et al., 2020).

## 4 Results and discussion

The free swell index test was used to evaluate the performance of raw and CMC-modified bentonite in the presence of the synthetic leachates with different cations concentrations, which were suggested by RSM software, including Cu^2+^ and Zn^2+^, and Cu^2+^/Zn^2+^.

### Solution including one cation

**Figure 3:**
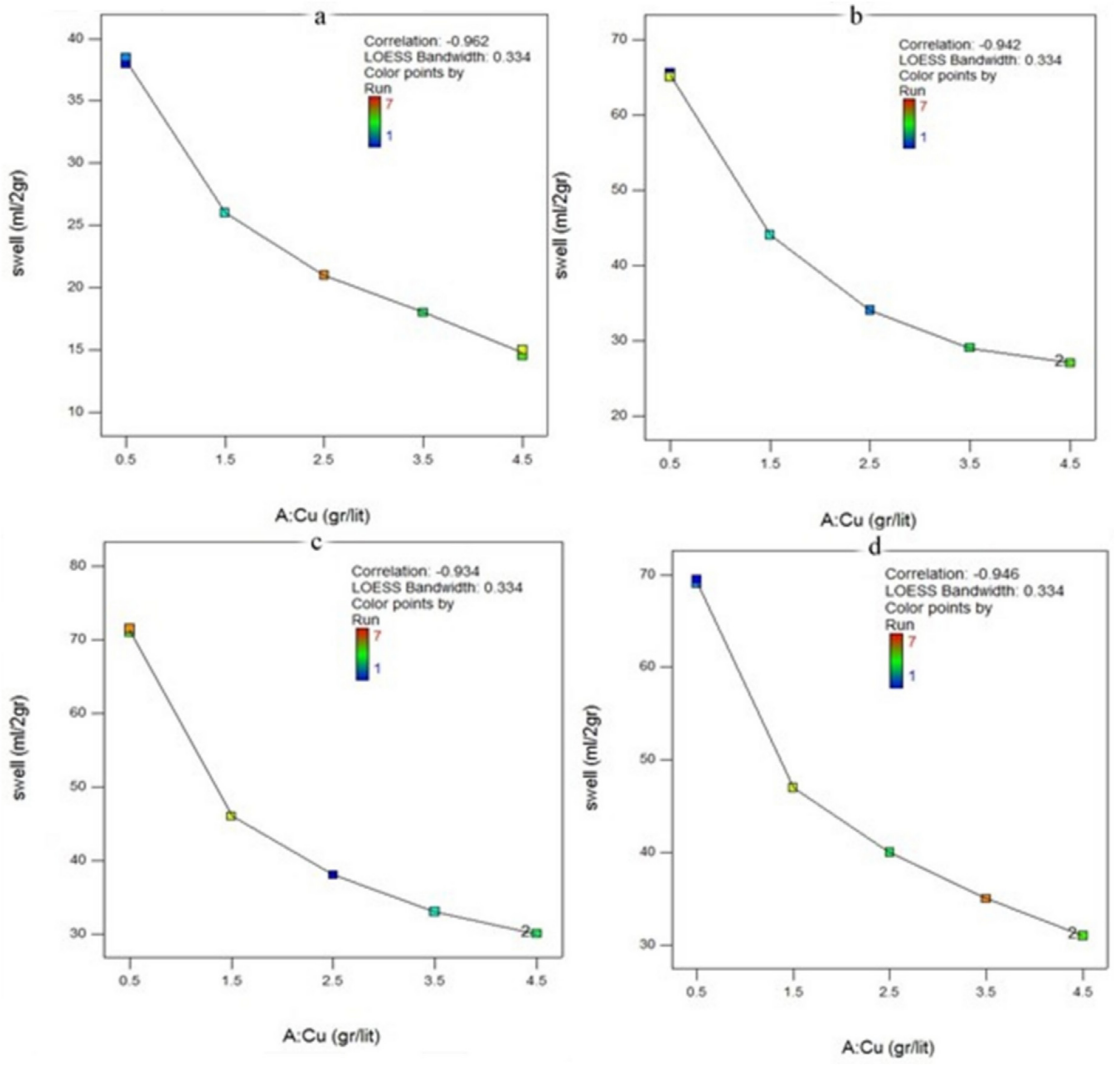
The free swelling of bentonite in the presence of a solution containing copper a) natural bentonite b) modified bentonite with 8% CMC c) modified bentonite with 10% CMC d) modified bentonite with 12% CMC.

**Figure 4:**
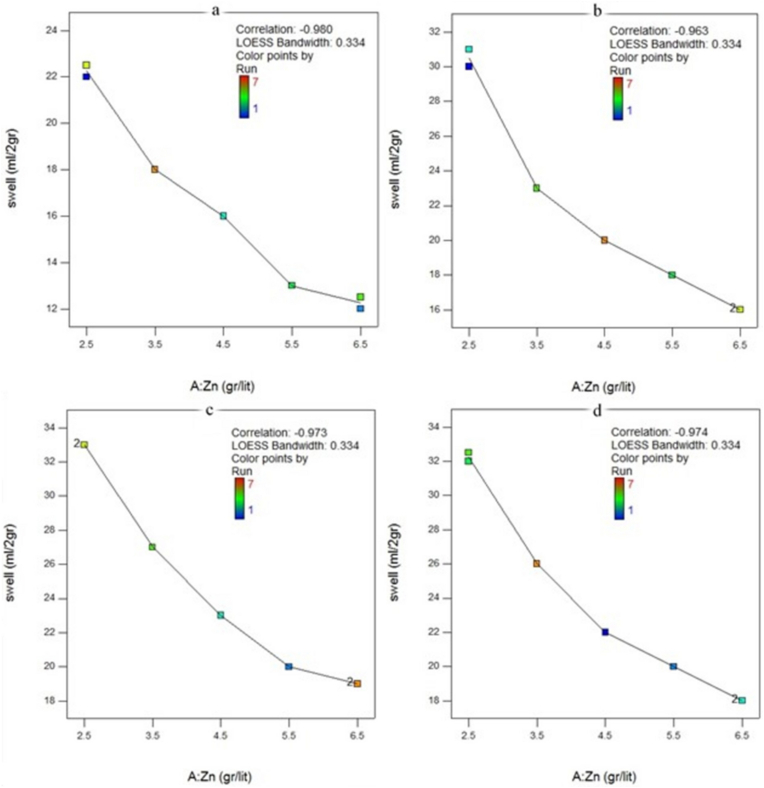
The free swelling of bentonite in the presence of a solution containing Zinc a) natural bentonite b) modified bentonite with 8% CMC c) modified bentonite with 10% CMC d) modified bentonite with 12% CMC.

Figures 3 and 4 show the free swell index (SI) of untreated and CMC-modified bentonite in the presence of solutions, including different concentrations of copper and zinc, separately. The results revealed that the swell index of both bentonite and modified bentonite decreased with an increase in the ion concentrations of the solutions.

**Figure 5:**
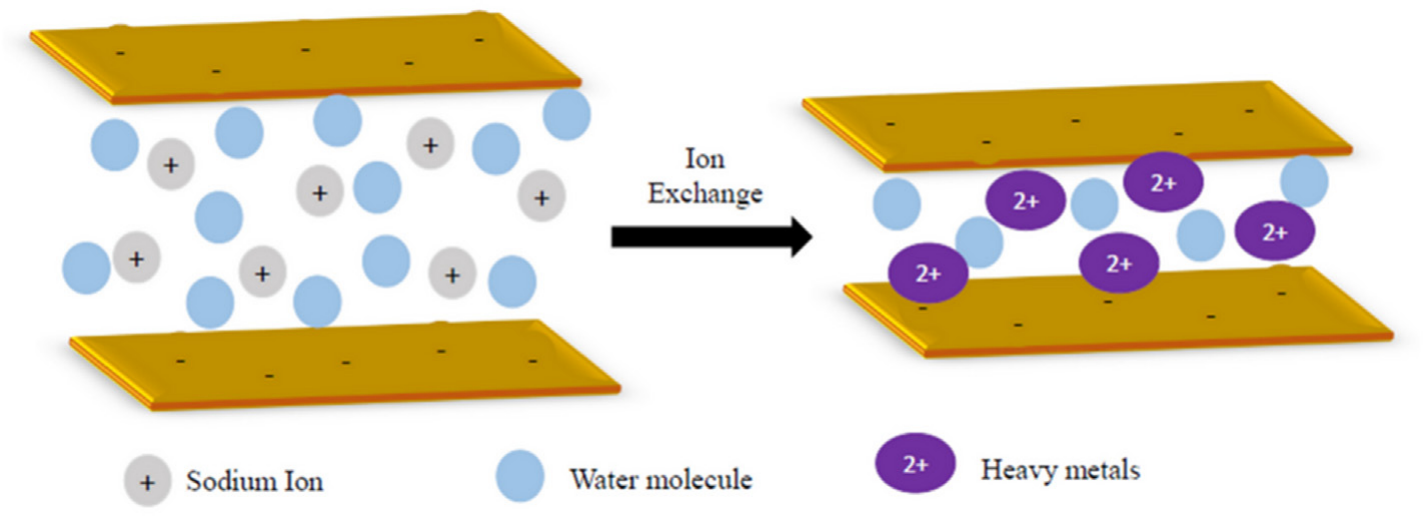
Ion exchange of monovalent sodium ions against heavy metals in bentonite.
2) the trapped cations between the negative clay layers.

This reduction in the swell index, which is related to the DDL of the bentonite, is due to two processes (Ashmawy et al., 2002):

1) the cation exchange between pollutants and the dominant cations of the bentonite particles, such as sodium and calcium ions, in the solution. This process can be seen in Figure 5.

Yet, there was a difference in the swell index against solutions containing Cu^2+^ and Zn^2+^. Zn^2+^ solutions decreased the swelling rate of the modified bentonite against mono-cationic solutions more than Cu^2+^ with the same molar concentrations. It can be said that Zn^2+^ had more tendency to participate in the cation exchange or be trapped between the bentonite layers in the modified bentonites. In contrast, the swell index of the natural bentonite exposed to the solutions, including Cu^2+^, was lower than Zn^2+^. Figure 4 illustrates that the GCL's performance against Zn^2+^ could improve by adding the proposed polymer. In other words, more Zn^2+^ ions may be trapped in the modified bentonite layers compared with the raw GCLs.

**Figure 6:**
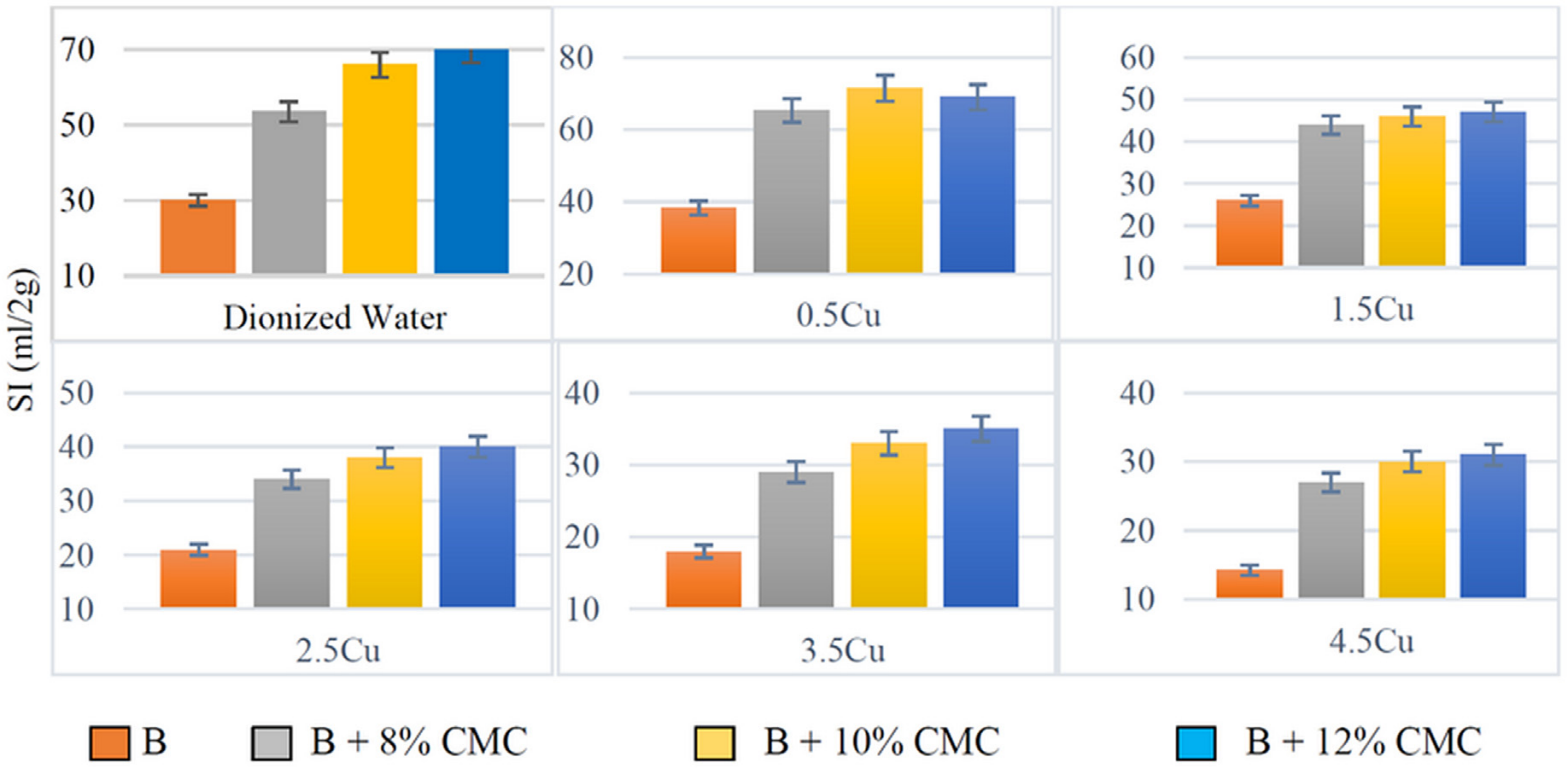
Effect of CMC adsorbent on each of the different concentrations of salt containing Cu^2+^ ions in terms of grams and distilled water in bentonite (B).

**Figure 7:**
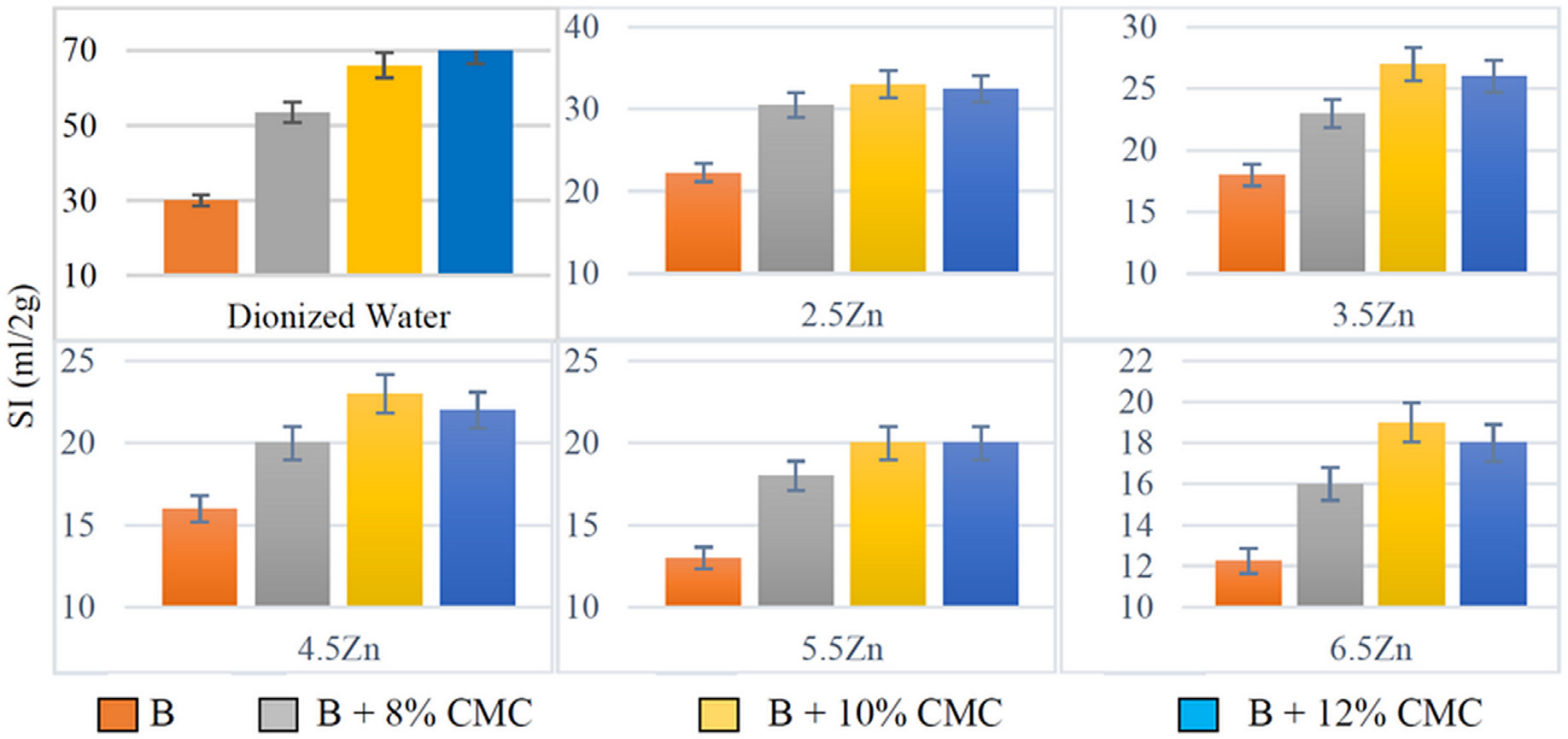
Effect of CMC adsorbent on each of the different concentrations of salt containing Zn^2+^ ions in terms of grams and distilled water in bentonite (B).

Figures 6 and 7 show the relationship between swelling and polymer content for each concentration used in this study. The SI of the modified bentonite was significantly higher than the swelling rate of the untreated bentonite in the presence of the same solution. Moreover, according to the acceptable range for the swell index, which is considered 25 or higher ml/2g (Ashmawy et al., 2002), CMC-modified bentonite could almost keep the swell index in this range while exposed to solutions with higher ion concentrations compared with raw bentonite. The reason for this positive effect of this polymer is the carboxylate (COO-) group, which has the ability to react with heavy metals, in its structure. Furthermore, when the ionic concentration of the solution exceeds 10 mmol/L, the ability of the polymer to preserve the high swelling rate decreases rather than in solutions with <10 mmol/L (Fan et al., 2020).

**Figure 8:**
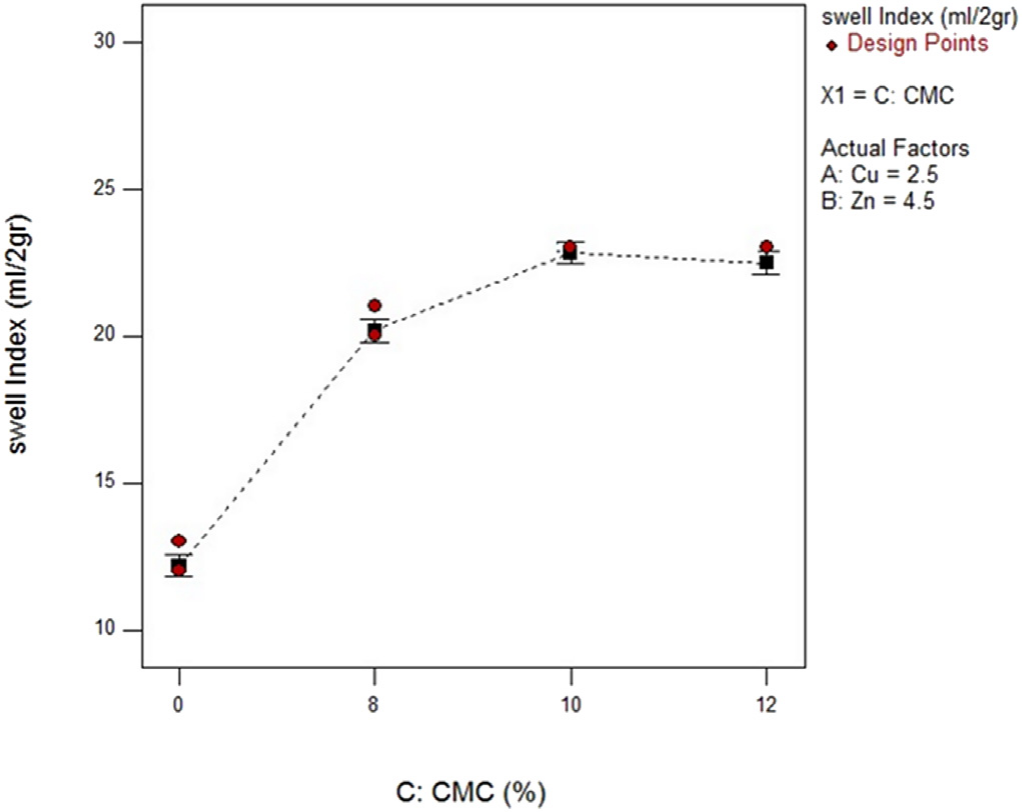
The total outline of the relationships between CMC adsorbent percentage and bentonite swelling behavior in ml/2g.

In total with increasing CMC, the swell index of the bentonite against deionized water and other heavy metals solutions increased and this change is due to the bond between the anionic polymer chain and the negative clay minerals, which not only causes an increase in the distance between the bentonite layers but also more areas in the bentonite are created to trap more pollutants (Fan et al., 2020; Kaoser et al., 2004b). In addition, modified bentonite with 10% CMC had overall better performance in solutions with higher concentrations. Figure 8, which was obtained from the RSM, presents the total outline of the influence of CMC on the swell index of the GCL. In detail, the bentonite swell index correlated directly with the CMC amount ranging from 0% to 10%. It follows that a slightly decreasing trend was seen in the swelling rate of modified bentonite with 12% CMC compared with modified bentonite with 10% CMC. Therefore, the optimum amount of this anionic polymer for the bentonite of GCL in the presence of the heavy metals solutions containing copper and zinc was 10%, which is in good agreement with the literature that has been conducted on the other pollutants’ adsorption by CMC-clays, which were modified with this polymer (Ri-Dong Fan et al., 2020; Janssen et al., 2015; Ma et al., 2019).

### Solution including two cations

In this section, the results regarding the performance of natural bentonite and CMC-modified bentonite against two-cation solutions containing copper and zinc are shown. In fact, there is competition among the heavy metals in the multi-cation solutions to participate in the cation exchange with bentonite. The cations' properties affect the GCLs’ performance, including the valence, the hydration radius of the ion, and the presence of other elements in the solutions (Pandey et al., 2019). The ion charge has a direct correlation with its adsorption rate. If the cations in the passing leachate have the same charge, the hydration radius of the ion and the effect of another metal can determine the swell index of the bentonite (Jo et al., 2001; Kaoser et al., 2004b).

**Figure 9:**
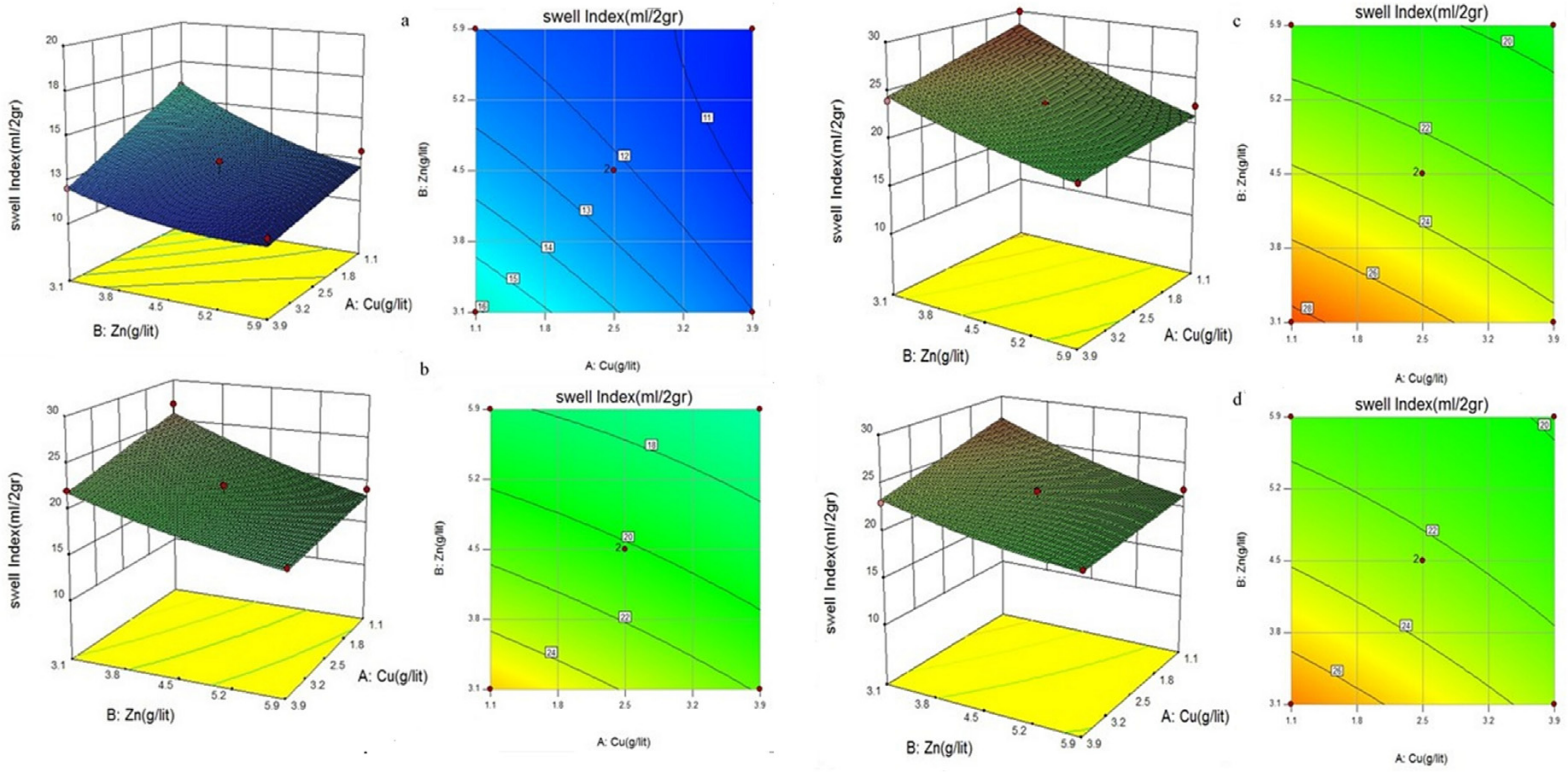
Effect of copper and zinc salts simultaneously on the swelling performance of a) Natural bentonite and modified bentonite with b) 8% CMC c) 10% CMC d) 12% CMC.

Figure 9, which was obtained from the RSM, includes both 3D and surface procedure diagrams, in order to demonstrate the simultaneous effects of Cu^2+^ and Zn^2+^ on the swell index of both unmodified and CMC-modified bentonites. In addition, the line slope of the surface procedure diagrams showed the effects of these two heavy metals on each other in the bentonites. In fact, this diagram defined which ion had more power to influence the swell index rather than another one.

In detail, it can be seen in the 3D diagrams that the swell index of both raw and CMC-modified bentonites decreased with an increase in the ion concentrations of the passing solutions. Moreover, it is cited that the polymer addition could improve the swelling potential of bentonite against high ionic concentrations of leachate. For instance, to change the swell index of the raw bentonite, an increase in Cu^2+^ concentrations was similar to that in Zn^2+^ concentrations. While this matter differed by adding the polymer to the bentonite. Whereas, 10% CMC-modified bentonite was the optimum mixture for the barrier exposed to these two heavy metals solutions. It can be noted that the free swell index of both raw and CMC-modified bentonites against solutions, containing Cu^2+^/Zn^2+^ was more sensitive to the changes in Zn^2+^ concentration compared with the changes in the concentration of Cu^2+^ by comparing the slope of the swell index-Zn^2+^ and the swell index-Cu^2+^ lines. The obtained surface procedure diagrams can prove this result.

In detail, the effect of the changes in Zn^2+^ concentration on the free swell index of bentonite was almost twice that of the changes in Cu^2+^ concentration. It means that the swell index of raw and CMC-modified bentonites was more susceptible to Zn^2+^ ascent than Cu^2+^ ascent. For example, in 8% CMC-bentonite against Cu^2+^/Zn^2+^, if Zn^2+^ concentration increased by approximately 0.7 g/l, Cu^2+^ concentrations should increase by 1.4 g/l in order to yield the same swell index. This result was consistent with the findings of Dutta and Mishra (2016), who discovered that the presence of Zn^2+^ in two cationic solutions could affect the drop in Liquid Limit (LL) and Plasticity Index (PI), which determine clay properties, twice as much as Cu^2+^. In total, a decrease in the Liquid Limit (LL) and Plasticity Index (PI) of clays show poorer barrier function against leachate because a decrease in the LL and PI can decrease the swelling rate of the bentonites of GCLs. This matter could be related to the negative effect of Zn^2+^ on the water adsorption of bentonite because (Nartowska, 2019) stated that an increase in the concentration of Zn^2+^ caused a significant reduction in the water adsorption of bentonite. On the other hand, Zn^2+^, which has less hydration radius than Cu^2+^, affects the swell index of the bentonite more than Cu^2+^ because the hydration radius of the ion has an inverse effect on the GCLs’ performance (Jo et al., 2001; Kaoser et al., 2004b; Nartowska, 2019).

**Figure 10:**
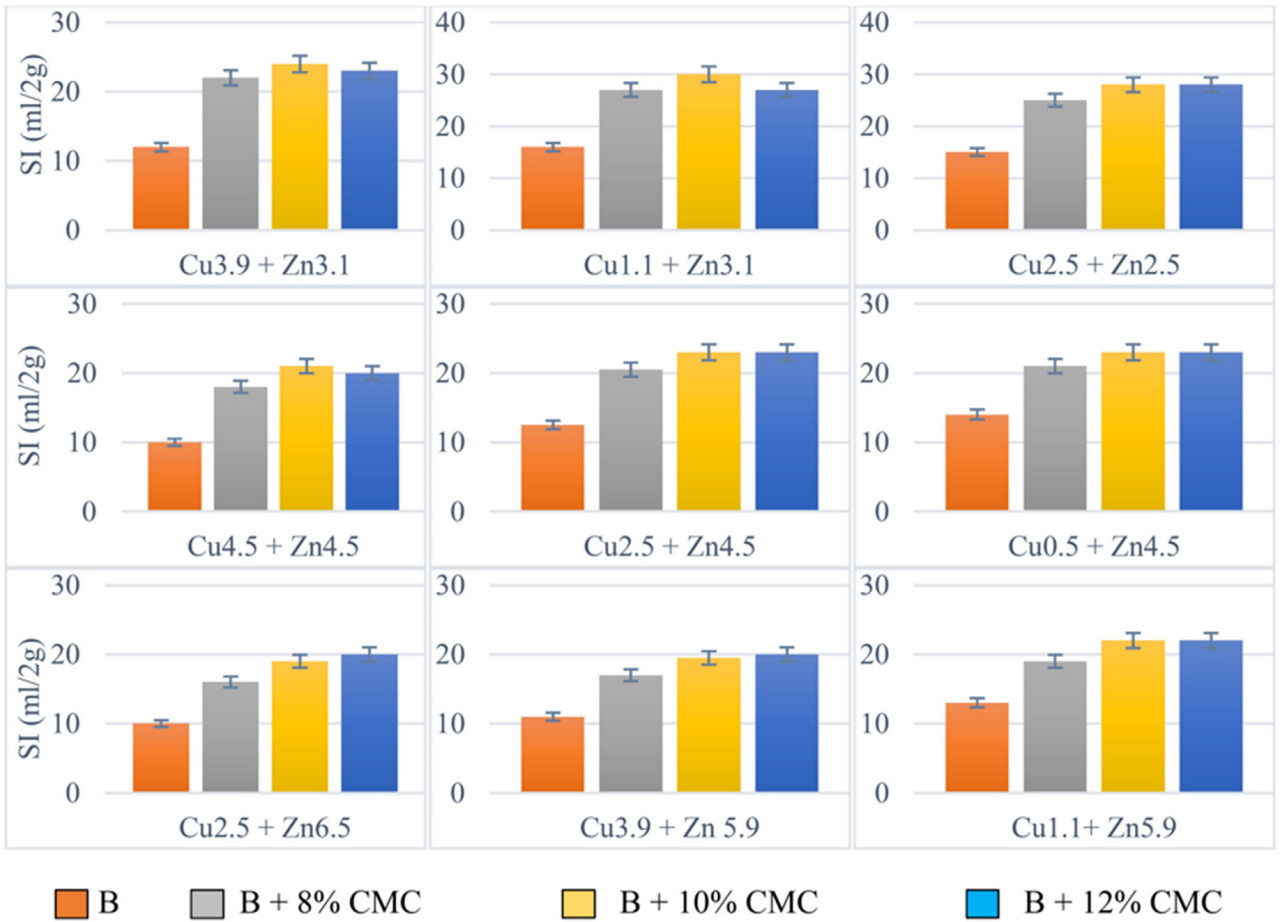
Effect of CMC adsorbent on Cu–Zn dual cation solution in terms of gram and distilled water in bentonite (B).

Considering the effect of the polymer content on the swelling performance of the bentonites in the presence of two cationic solutions with different concentrations, Figure 10 shows that modified bentonite with 10% CMC had overall the best performance in the presence of the multi-cationic electrolyte solutions.

### Scanning Electron Microscopy

**Figure 11:**
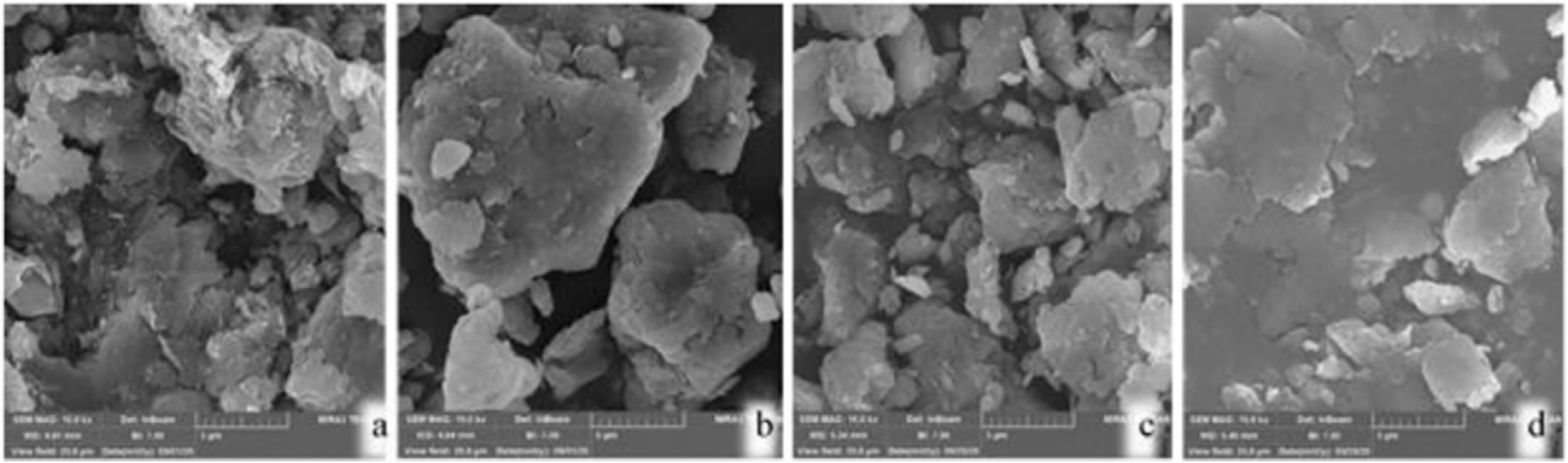
SEM image a) Natural bentonite b) Modified bentonite with 8% CMC c) Modified bentonite with 10% CMC d) Modified bentonite with 12% CMC.

As said before, CMC polymers ascended the distance between the negative bentonite layers due to their negative valence. SEM pictures can confirm these positive effects of the polymer content on the bentonite properties, such as the DDL and the specific surface area (SSA). As shown in Figure 11, the distances between the bentonite layers, which are depicted by the dark areas, increased with an increase in the polymer content. As a result, the high swell index, as one of the major parameters for the GCL performance evaluation, can be achieved. It can also be seen that the bright areas, which are related to the specific surface of the bentonite, increased with an increase in the polymer content. It follows that more spots are created to trap heavy metals in the bentonite layers while increasing the SSA. In total, 10% CMC-modified bentonite had a greater SSA compared with others.

## 5 Conclusion

In this research, the swelling rate of the natural bentonite and CMC-modified bentonite exposed to the solutions containing heavy metals, such as Cu^2+^ and Zn^2+^, separately and simultaneously, were investigated. The following results could be obtained:

• CMC, the anionic polymer, could increase the bentonite swell index against deionized water and the electrolyte solutions, including heavy metals due to an increase in the distance between the bentonite layers. For example, a 233% increase in 12% CMC-modified bentonite was seen compared with the natural bentonite against deionized water.
• The swelling potential of the bentonites decreases while increasing the ion concentrations of the permeant solutions because increasing the ion concentrations causes an increase in the adsorption amount and the cation exchange capacity with the bentonites. It follows that the distance between the layers in the bentonites decreases. In addition, the results showed that the swell index of the natural bentonite exposed to Cu^2+^ decreased, which accumulated to about 50%. While the swelling rate of the natural bentonite against Zn^2+^ (with similar molar concentration) decreased, accounting for about 40%. This data represented that Zn^2+^ had a lower tendency to react with the raw bentonite compared with Cu^2+^. Thus, the solutions, including zinc, had a lower effect on the swell index of the natural bentonite. On the other hand, the decreasing trend had also been observed for the CMC-modified bentonite against copper and zinc, accumulating to approximately 50% and 60% decrease (on average), respectively. This matter demonstrated that the polymer addition could improve the reaction of Zn^2+^ with the bentonite. While the Cu^2+^ adsorption rate was fixed. It means that the swelling rate of the modified bentonite against Zn^2+^ experienced a 20% decrease more than that of the natural bentonite. Thus, it can be said that the addition of this polymer could prevent Zn^2+^ entering into the soil and groundwater more than the raw GCL.
• In the solutions containing these two cations, simultaneously, competition is created between the cations to participate in the cation exchange with bentonite, in which the amount of ion charge and its hydrated radius are the influential factors.
• The free swell index of the natural and modified bentonite against the binary solutions containing heavy metals of copper and zinc was more sensitive to the zinc metal concentration compared with the copper concentrations due to the lower hydration radius of Zn^2+^ than Cu^2+^. It means, if the concentration of Cu^2+^ in solution was assumed to be constant, an increase in Zn^2+^ concentrations had slightly more influence on the decreasing trend in the swell index of the bentonite and modified bentonite than the inverse situation.
• Despite the increase in the swell index of the bentonite with increasing CMC, 10% CMC-modified bentonite showed the better overall barrier performance against deionized water and the solutions with one or two heavy metals derived from E-waste leachate.

The findings of the swell index tests in this study suggested that 10% CMC-modified bentonite of GCL had a noticeable improvement in the GCLs' performance when subjected to the synthetic E-waste solutions, including Cu^2+^ and Zn^2+^, separately and simultaneously, rather than the natural GCLs. Considering this issue that the behavior of GCL exposed to the leachate with high concentrations of heavy metals is the main concern of industries and researchers, the permeability of the GCL should be investigated against other synthetic solutions and real E-waste leachates in order to be assured of the effective application of this modified GCL as the safer barrier and to limit the risk of entering leachate into the soil and groundwater. In addition, to evaluate GCLs’ lifetime or their function under different environmental conditions as the leachate barriers, the long-term hydraulic conductivity against synthetic and real leachates should be analyzed.

## Declarations

### Author contribution statement

Maryam Roshan Mooshaee: Conceived and designed the experiments; Performed the experiments; Analyzed and interpreted the data; Contributed reagents, materials, analysis tools or data; Wrote the paper.

MohammadReza Sabour: Conceived and designed the experiments; Analyzed and interpreted the data.

Ebad Kamza: Conceived and designed the experiments; Analyzed and interpreted the data; Contributed reagents, materials, analysis tools or data.

### Funding statement

This research did not receive any specific grant from funding agencies in the public, commercial, or not-for-profit sectors.

### Data availability statement

Data will be made available on request.

### Declaration of interests statement

The authors declare no competing interests.

### Additional information

No additional information is available for this paper.
